# Effects of Combined Spinal-Epidural Analgesia during Labor on Postpartum Electrophysiological Function of Maternal Pelvic Floor Muscle: A Randomized Controlled Trial

**DOI:** 10.1371/journal.pone.0137267

**Published:** 2015-09-04

**Authors:** Ji-Juan Xing, Xiu-Fen Liu, Xiao-Ming Xiong, Li Huang, Cheng-Yi Lao, Mei Yang, Shan Gao, Qiong-Yan Huang, Wei Yang, Yun-Feng Zhu, Di-Hua Zhang

**Affiliations:** 1 Anesthesia Department, Maternal and Child Health Hospital of Nanning City, Nanning 530011, China; 2 Pain Management Department, First Affiliated Hospital of Peking University, Beijing 100034, China; 3 Obstetrics and Gynecology Department, Maternal and Child Health Hospital of Nandan County, Hechi 547200, China; 4 Maternity Care Department, Maternal and Child Health Hospital of Nanning City, Nanning 530011, China; 5 Child Care Department, Maternal and Child Health Hospital of Nanning City, Nanning 530011, China; Oslo University Hospital, Ullevål, NORWAY

## Abstract

**Objective:**

Combined spinal-epidural analgesia (CSEA) is sometimes used for difficult births, but whether it contributes to postpartum pelvic muscle disorder is unclear. This randomized controlled trial examined whether CSEA given during labor affects the electrophysiological index of postpartum pelvic floor muscle function.

**Methods:**

A consecutive sample of primiparous women who delivered vaginally at term were randomly assigned to a CSEA group (n = 143) and control group (n = 142) between June 2013 and June 2014. All were assessed 6–8 weeks later for electrophysiological function of pelvic floor muscle.

**Results:**

The two groups were similar in the degree of muscle strength, muscle fatigue, and pelvic dynamic pressure of pelvic floor muscle. The CSEA and control groups showed similar proportions of women with normal muscle strength (score ≥4) in type I pelvic fibers (23.1% vs. 14.1%, *P* = 0.051) and type II pelvic fibers (28.0% vs. 24.6%, *P* = 0.524). The groups also contained similar proportions of women who showed no fatigue in type I fibers (54.5% vs. 48.6%, *P* = 0.315) or type II fibers (88.8% vs. 87.3%, *P* = 0.699). Similarly low proportions of women in the CSEA group and control group showed normal pelvic dynamic pressure (11.2% vs. 7.7%, *P* = 0.321). However, women in the CSEA group spent significantly less time in labor than those in the control group (7.25 vs. 9.52 h, *P* <0.001).

**Conclusions:**

CSEA did not affect the risk of postpartum pelvic muscle disorder in this cohort of primiparous women who gave birth vaginally. A significant shorter duration of labour was observed in the CSEA-group.

**Trial Registration:**

ClinicalTrials.gov NCT02334150

## Introduction

Labor analgesia can relieve labor pain, reduce stress reactions, and improve blood supply to the fetus, benefiting mother and baby [1–3]. Though traditional epidural analgesia has been used for more than 40 years, combined spinal-epidural analgesia (CSEA) has become popular because it provides faster-onset pain relief with minimal motor weakness [4]. CSEA may also accelerate cervical dilation [5].

Despite the popularity of CSEA, whether it is associated with short- or long-term beneficial or adverse effects for the mothers remains unclear. One question is whether the procedure affects the risk of female pelvic floor disorder (PFD), in which the pelvic floor muscles are injured. These muscles are responsible for supporting the pelvic organs and for stabilizing them during the rhythmic, strong labor contractions and for the diaphragm to contract enough to generate pressures of up to 19 kPa. Numerous risk factors have been associated with PFD, including obesity, diabetes, older age, connective tissue disorder, neurological disease, pregnancy, vaginal delivery and childbirth [6–8]. PFD may lead to stress urinary incontinence, overactive bladder, pelvic organ prolapse and fecal incontinence, all of which can strongly reduce women's physical and psychological health [6–8].

Pelvic floor function can be analyzed by measuring the strength and degree of fatigue of pelvic floor muscles, as well as the pelvic dynamic pressure. Abnormalities in these indicators appear even before patients complain of the signs and symptoms of PFD, making them a useful early diagnostic index [9].

In this randomized controlled study, we examined whether CSEA affects postpartum pelvic floor muscle function in primiparous mothers who give birth vaginally, as well as the duration of different stages of labor. By this study we aimed to contribute in the evaluation of whether the widespread use of CSEA provides benefits to mothers or poses a risk.

## Methods

### Patients

This randomized controlled trial protocol was approved by the Ethics Committee of the Maternal and Child Health Hospital of Nanning, China. Written consent was obtained from the mothers. We assigned the participants to either the treatment or control group using sealed envelopes. This study is followed and fulfilled the CONSORT criteria [10]. Our ethics committee approved the study at April 12, 2013 (S1 and S2 Protocol).When we started the trial, we were not aware of the importance of trial registration. So we registered the trial when the study has been completed in the clinicaltrials.gov at https://clinicaltrials.gov/ct2/show/NCT02334150. The authors confirm that all ongoing and related trials for this drug/intervention are registered. The period of follow-up was at least 3 months (12 weeks). Labor duration was not a primary outcome in our study protocol. However, this was later added as a secondary outcome.

### Inclusion criteria

Primiparous women who gave birth by vaginal delivery between June 2013 and June 2014 in the Maternal and Child Health Hospital of Nanning were eligible for inclusion in this trial. They would be asked to take part in this trial when the gestational age was between 38 and 40 weeks. They were enrolled if they (1) were 22–30 years old; (2) were 155–165 cm tall; (3) were assigned a score of I or II on the American Society of Anesthesiologists scale [11]; and (4) gave birth by vaginal delivery to a live, single, mature fetus (≥ 38, ≤ 40 w) in the vertex position with (5) a neonatal weight of 2900–3500 g.

### Exclusion criteria

Subjects were excluded if they had (1) history of chronic cough; (2) chronic constipation or pelvic organ resection; (3) family history of urinary incontinence; (4) pelvic organ prolapse; (5) any systemic disease before delivery; or (6) a history of surgery, trauma, tumor or deformity of lumbar vertebrae.

### Labor analgesia

Women in the CSEA group received CSEA during labor. An intravenous line was established when the cervical opening measured 1–2 cm. Then sufentanil (5–7 μg) was injected intrathecally. When the visual analogue pain score was 3 or higher, a mixture of ropivocaine (0.143%) and sufentanil (0.3 μg/ml) was continuously infused into the epidural space using an analgesia pump until the cervix was fully dilated [12]. Load capacity was 5 ml. The analgesic plane was controlled under T_10_.

Women in the control group were not provided any analgesia during labor.

### Electrophysiological outcomes

Pelvic floor muscle strength, degree of pelvic floor muscle fatigue and pelvic floor dynamic pressure were measured in the participating women of both groups on a single occasion 6–8 weeks after delivery. These tests were performed by a single experienced researcher using a PHENIX neuro-muscle stimulator (Vivalns, Paris, France) [13–14]. The researcher was blinded to the mothers' group allocations. The acceptable range of air inflation volume, defined as the volume added before the woman reported feeling vaginal fullness, was 15–30 ml. Women who reported fullness with a volume <15 ml or >30 ml were excluded from the study.

The international muscle strength measurement method used in this study has been validated in several previous studies [15–17]. Pelvic floor muscle strength was classified according to the Oxford scale [17]. The postpartum strength of pelvic floor muscles was measured with subjects in the supine lithotomy position. A vaginal manometric probe with a balloon was sheathed with a condom and placed into the vagina; the top of the device lay at the bottom of the vagina before air inflation. The other side of the probe was connected to the PHENIX muscle stimulator. Muscle strength was assessed using the international muscle strength detection method, which features a strength scale from 0 to 5 [18]. The strength score depends on whether vaginal muscle contraction upon stimulation fails to occur (0 points) or lasts 1 second (1 point), 2 seconds (2 points) and so on. If the contraction lasts 5 seconds or more, a strength score of 5 is assigned.

Pelvic floor muscle strength was considered normal if both type I and II muscle fibers received a score of 4 or 5. Degree of muscle fatigue was considered normal if no fatigue was noted, thus a result of-1 to-3 was considered abnormal. Pelvic floor dynamic pressure was considered normal when measured within the range of 80 and 150 cm H_2_O.

Before the measurement, participants were taught how to perform a correct pelvic floor muscle contraction. The instructions of pelvic floor rehabilitation after delivery have been described in previous studies [19–21].

### Statistical analysis

Continuous data were expressed as median (range). The statistical significance of differences in continuous data was analyzed using the Mann-Whitney U test, and the significance of differences between categorical data was assessed with the chi-squared test. When several cells have expected numbers <5, Fisher´s exact test was used. For all tests, a two-tailed P value <0.05 was considered statistically significant. All statistical analyses were performed using the SPSS 17.0 statistical package (SPSS, Chicago, Illinois).

## Results

### Characteristics

**Fig 1:**
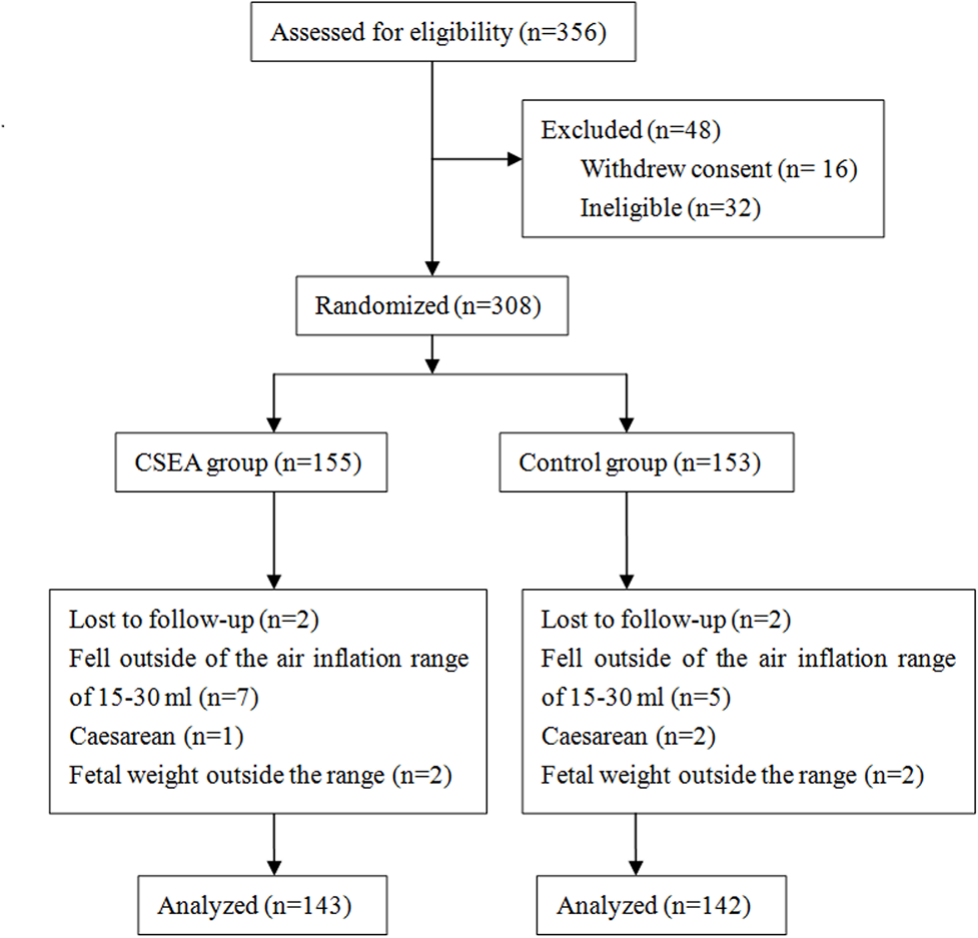
CONSORT flow diagram for randomized controlled study comparing postpartum electrophysiologic pelvic floor measurements after spontaneous vaginal delivery with combined spinal-epidural analgesia (CSEA) or no analgesia.

During the recruitment period, 356 women were eligible to enroll in our study, but 48 were excluded because they did not satisfy the inclusion criteria or they withdrew consent. Another 12 women were excluded because they reported feeling vaginal fullness at an inflation volume <15 ml or >30 ml. Another 4 were excluded after being lost to follow-up. Three were delivered by caesarean and 4 fetal weight were outside the range of 2900–3500 g. These 7 women were also excluded. All deliveries started spontaneously and none received labor augmentation, such as oxytocin. Moreover, forceps and ventouse were not used. Five women who were allocated to control group requested CSEA. However, all the data of these five women were kept in control group (according to the intention to treat principle). In total, 143 women were included in the CSEA group and 142 in the control group (Fig 1). The two groups showed no significant differences in maternal age, height, weight, or gestational age, newborn weight or babies sex, and perineal and/or sphincter laceration (Table 1).

**Table 1:** Baseline characteristics of newborn and primiparous women who received combined spinal-epidural analgesia during labor or no analgesia at all.

Characteristic	CSEA group (n = 143)	Control group (n = 142)	*P* value
Age of mother, median (range), yr	26.4 (22–30)	26.5 (22–30)	0.156*
Height of mother, median (range), cm	159.8 (155–165)	160.4 (155–165)	0.359*
Weight of mother, median (range), kg	51 (40–82)	50 (42–84)	0.329*
Gestational age, median (range), wk	39.2 (38–40)	38.9 (38–40)	0.428*
Newborn weight, median (range), g	3 248 (2906–3490)	3 252 (2915–3489)	0.403*
Babies sex, M/F	69/74	78/64	0.259**
Time point for the electrophysiologic measure, median (range), d	48 (42–56)	50 (42–56)	0.517*
Perineal and/or sphincter laceration	20	23	0.551**

CSEA, combined spinal-epidural analgesia; d, day; F, female; M, male; wk, week; yr, year.

* Mann-Whitney U test.

** Chi-squared test.

### Electrophysiological outcomes

Electrophysiology analysis 6–8 weeks after delivery showed no significant differences between the groups in the distribution of muscle strength scores for type I or type II fibers (Table 2), in the distribution of degrees of muscle fatigue (Table 3), or in the distribution of pelvic floor dynamic pressures (Table 4).

**Table 2:** Distribution of pelvic floor muscle strength scores in primiparous women who received combined spinal-epidural analgesia during labor (n = 143) or no analgesia at all (n = 142).

Muscle strength	Type I muscle fibers*		Overall *P* value**	Type II muscle fibers*		Overall *P* value**
	CSEA	Control		CSEA	Control	
0	95 (66.4)	104 (73.2)	0.115	70 (49.0)	74 (52.1)	0.409
1	9 (63)	14 (9.9)		11 (7.7)	16 (11.3)	
2	2 (1.4)	2 (1.4)		10 (7.0)	9 (6.3)	
3	5 (3.5)	3 (2.1)		12 (8.4)	9 (6.3)	
4	5 (3.5)	4 (2.8)		9 (6.3)	8 (5.6)	
5	27 (18.9)	15 (10.6)		31 (21.7)	26 (18.3)	

CSEA, combined spinal-epidural analgesia.

* Values indicate the number of women in each group (and % of total) who received the indicated strength score.

** Mann-Whitney U test.

**Table 3:** Distribution of pelvic floor muscle fatigue degrees in primiparous women who received combined spinal-epidural analgesia during labor (n = 143) or no analgesia at all (n = 142).

Degree of muscle fatigue	Type I muscle fiber*		Overall *P* value**	Type II muscle fibers*		Overall *P* value**
	CSEA	Control		CSEA	Control	
0	76 (53.1)	67 (47.2)	0.215	124 (86.7)	122 (85.9)	0.851
-1	42 (29.4)	41 (28.9)		16 (11.2)	17 (12.0)	
-2	19 (13.3)	27 (19.0)		1 (0.7)	2 (1.4)	
-3	6 (4.2)	7 (4.9)		2 (1.4)	1 (0.7)	

CSEA, combined spinal-epidural analgesia.

*Values indicate the number of women in each group (and % of total) who were assigned the indicated fatigue degree.

** Mann-Whitney U test.

**Table 4:** Distribution of pelvic floor dynamic pressure values in primiparous women who received combined spinal-epidural analgesia during labor (n = 143) or no analgesia at all (n = 142).

Dynamic pressure (cmH_2_O)	No. of women (%)		*P* value
	CSEA	Control	
< 80	125 (87.4)	130 (91.5)	0.263*
80–150	16 (11.2)	10 (7.0)	
> 150	2 (1.4)	2 (1.4)	

CSEA, combined spinal-epidural analgesia.

* Mann-Whitney U test.

When measurements were categorized as normal or abnormal based on prevailing clinical standards, similar proportions of women in the CSEA and control groups showed normal dynamic pressure values and normal scores for pelvic muscle strength and fatigue for type I and II fibers (Table 5).

**Table 5:** Comparison of rates of normal pelvic dynamic pressure and normal electrophysiological function of pelvic floor muscle in primiparous women who received combined spinal-epidural analgesia during labor (n = 143) or no analgesia at all (n = 142).

Outcome	No. of women (%)		*P* value
	CSEA	Control	
Normal pelvic dynamic pressure, n (%)*	16 (11.2)	11 (7.7)	0.321****
Normal type I pelvic floor muscle strength, n (%)**	33 (23.1)	20 (14.1)	0.051****
Normal type II pelvic floor muscle strength, n (%)**	40 (28.0)	35 (24.6)	0.524****
Normal degree of type I pelvic floor muscle fatigue, n (%)***	78 (54.5)	69 (48.6)	0.315****
Normal degree of type I/type II pelvic floor muscle fatigue, n (%)***	127 (88.8)	124 (87.3)	0.699****

CSEA, combined spinal-epidural analgesia.

*Defined as 80–150 cmH_2_O.

**Defined as an International Muscle Strength Detection score of 4 or 5 for both type I and type II fibers.

***Defined as a fatigue degree of 0.

****Chi-squared test

### Duration of labor

Women of the CSEA group had significantly shorter first-stage and second-stage labor than the control group, as well as shorter total labor (Table 6).

**Table 6:** Comparison of the duration of different labor stages in primiparous women who received combined spinal-epidural analgesia during labor (n = 143) or no analgesia at all (n = 142).

Labor stage	CSEA group	Control group	*P* value
First stage, h	6.22 (3.17–9.27)	8.63 (5.37–11.89)	<0.001*
Second stage, min	46.19 (28.7–63.68)	51.76 (32.35–71.17)	0.011*
Total labor duration, h	7.25 (4.63–9.87)	9.52 (6.49–12.55)	<0.001*

CSEA, combined spinal-epidural analgesia; h, hour; min, minutes.

* Mann-Whitney U test.

## Discussion

We have performed a small-scale randomized controlled study evaluating postpartum electrophysiological outcomes in women who gave birth vaginally without any analgesia or with CSEA using ropivocaine and sufentanil. Our results suggest that the method does not increase the risk of PFD up to 8 weeks after delivery, and that it may help shorten the two stages of labor, particularly the second stage, when the pelvic muscles must remain dilated to support rhythmic contractions, and the baby experiences a strong increase in pressure passing through the birth canal. Our findings suggest that the growing use of this form of labor analgesia is justified in regard of it not imposing greater risk for initial pelvic floor muscular dysfunction.

Our findings add to the published advantages of CSEA over traditional epidural techniques. Unlike the traditional epidural, CSEA does not increase the risk of labor augmentation therapy or instrumental vaginal delivery. CSEA is also believed to improve maternal mobility during labor and provide faster-onset analgesia than traditional epidural analgesia, thereby increasing maternal satisfaction and reducing the need for rescue analgesia [22].

Despite the medical care provided during delivery, obstetric and anal sphincter injuries are still prevalent and may lead to diverse PFD [23]. Previous studies have reported that CSEA increases the risk of PFD [24,25]. In contrast, our electrophysiological assessment of pelvic floor muscles 6–8 weeks after delivery showed similar distributions of muscle strength and fatigue in both the CSEA and control groups. In fact, when women were classified as having normal or abnormal pelvic outcomes, the CSEA group showed higher proportions of women with normal type I and II muscle strength (scores ≥4), without detected muscle fatigue and normal pelvic floor dynamic pressure (80–150 cmH_2_O) than the control group. These results suggest that CSEA may even show a slight benefit over no analgesia in women with complication-free pregnancies who undergo vaginal delivery. This may be linked to our finding that CSEA shortens first two stages of labor: shortening labor, particularly the key second stage, reduces the physical and psychological difficulties and the so-called fear-nervous-pain syndrome [26], in which nervousness and pain cause local inflammatory edema and stimulate the release of catecholamine, which suppresses effective and rhythmic uterine contractions.

Vaginal delivery has been associated with increased risk of postpartum stress incontinence [27]. In our study, fewer than 50% of CSEA and control women had normal type I and type II pelvic floor muscle strength and normal pelvic dynamic pressure. In addition, only half the women in both groups had a normal degree of type I pelvic floor muscle fatigue. Such low values for these three early diagnostic indicators of PFD suggest impairment of the pelvic floor. Nevertheless, the two groups showed similar rates of normal pelvic outcomes (Table 5). Our results support previous studies that vaginal delivery causes varying degrees of pelvic floor muscle injury. They further suggest that CSEA is not associated with increased risk of pelvic floor muscle injury as compared with no labor analgesia.

In addition to outcomes of pelvic floor muscle function, we measured the duration of the different stages of labor. We found women in the CSEA group experienced significantly duration of shorter first- and second-stage labor, as well as total labor. Risk of peripartum injury is closely related to labor duration, especially duration of the second stage of labor. Since shortening the second phase reduces the risk of postpartum PFD [28], CSEA may help reduce the risk of PFD. These effects of CSEA may help explain why it reduces the rate of cesarean sections [29].

Though this study provides the first controlled insights into the association between CSEA and the electrophysiological index of postpartum pelvic floor muscle, the results should be interpreted with caution. First, this study lacked a consistent double-blind design for each subject. Nevertheless, her attending nurse and physician attending at the electrophysiologic measurement procedure were blinded to the technique. Second, previous studies have reported that labor analgesia has side effects on the newborn [10,30,31], but we did not examine this question. Third, due to muscle function improve during months to come, electrophysiological outcome measurement on a single occasion of 6–8 weeks after delivery may be too early to access final pelvic muscle function, which may affect the real outcome measurement. Last, we did not perform sample size calculation before the trial began. Future controlled studies should solve these limitations and examine long-term effects of CSEA on both mothers and newborns.

In conclusion, this randomized controlled trial provides evidence that CSEA does not increase the risk of PFD, at least in the short term. CSEA also appears to shorten labor.

This randomized trial is guided by the CONSORT 2010 checklist (S1 CONSORT Checklist).

## Supporting Information

S1 CONSORT Checklist CONSORT 2010 checklist. (DOC)

S1 Protocol English version of clinical trial report protocol. (DOC)

S2 Protocol Chinese version of clinical trial report protocol. (DOC)
