# Supplementary figures and images for: Effects of Combined Spinal-Epidural Analgesia during Labor on Postpartum Electrophysiological Function of Maternal Pelvic Floor Muscle: A Randomized Controlled Trial
Source: PLoS One. 2015 Sep 4;10(9):e0137267. doi: 10.1371/journal.pone.0137267 (PMC4560435; doi:10.1371/journal.pone.0137267)

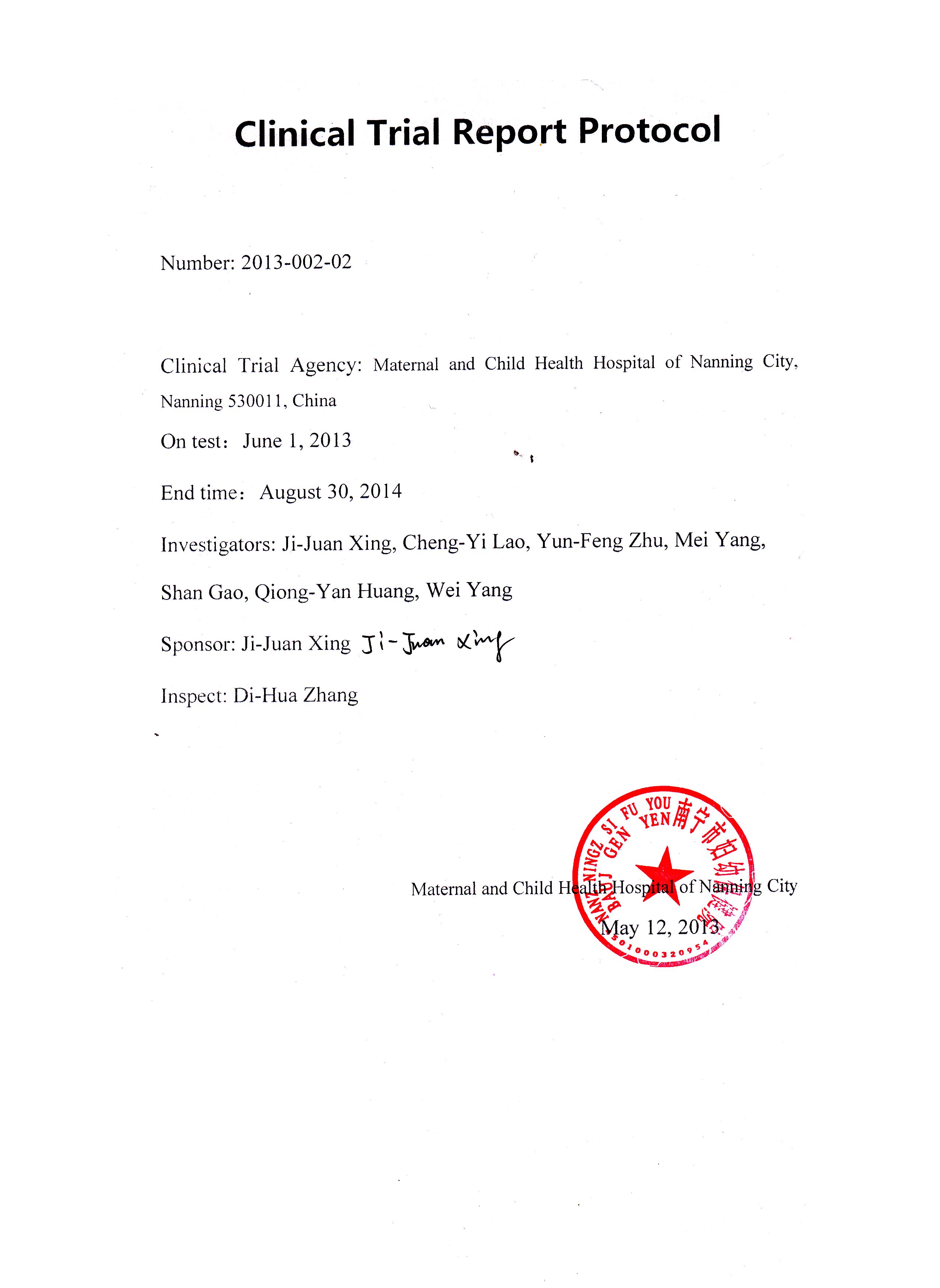


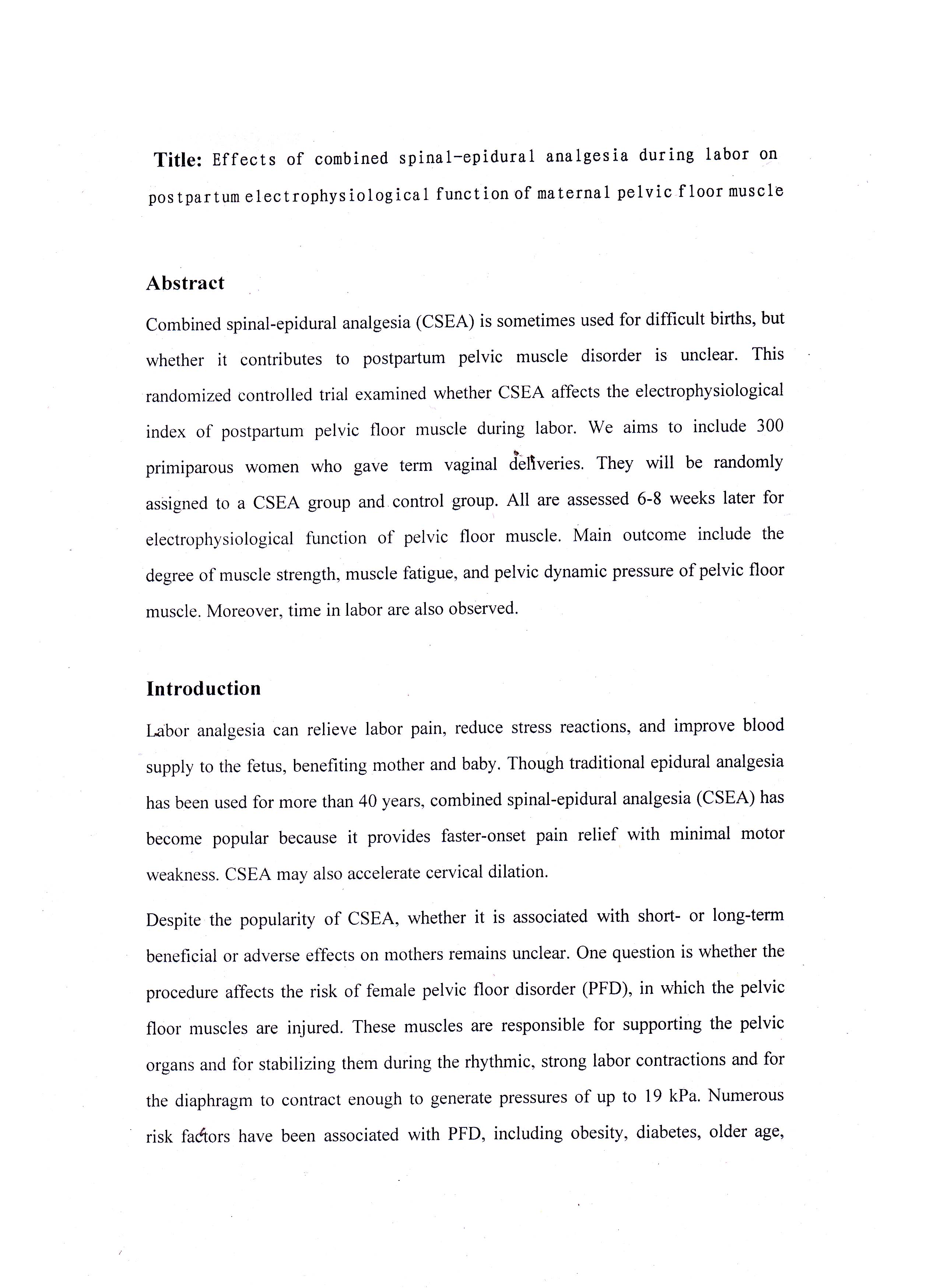


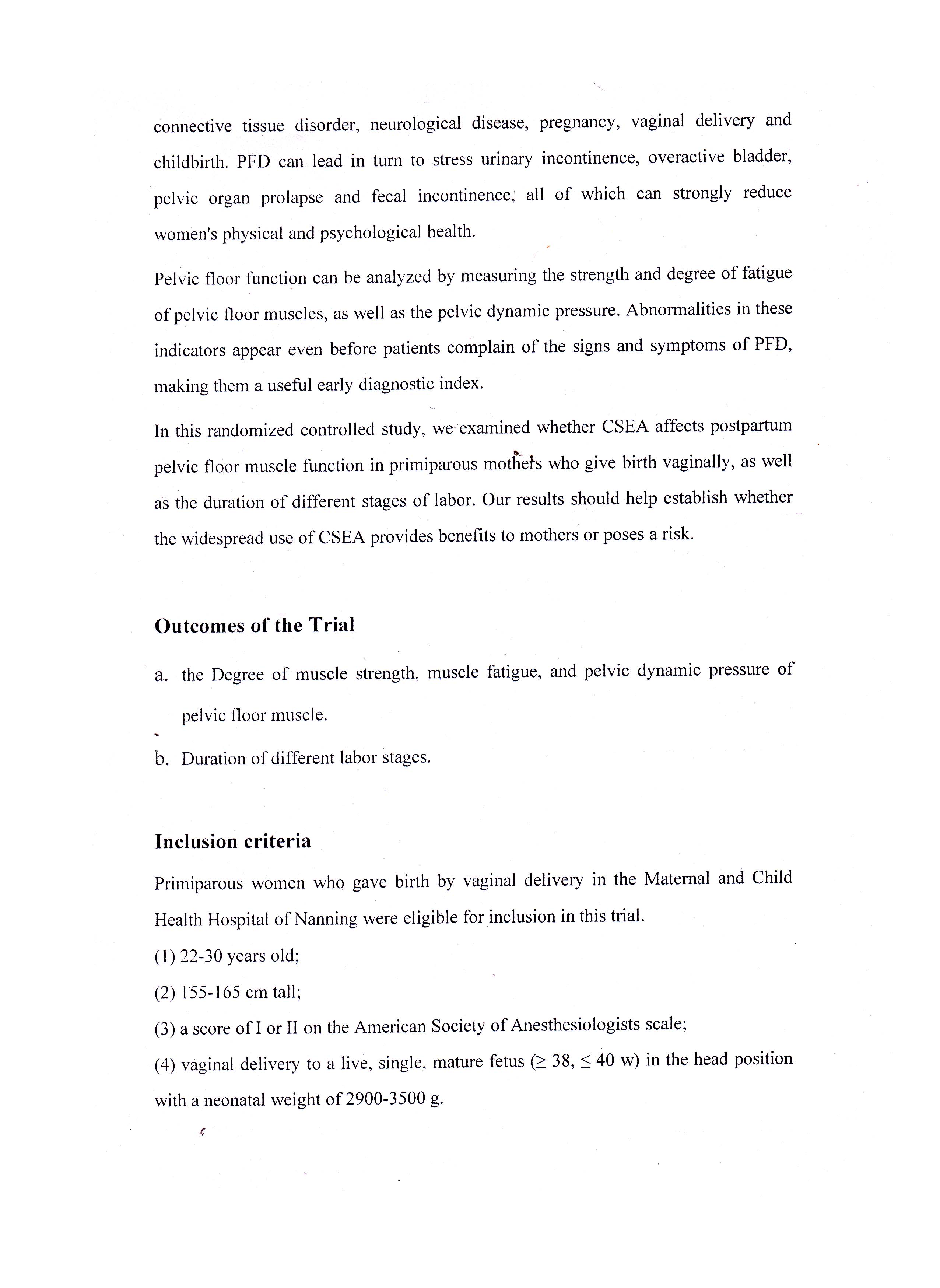


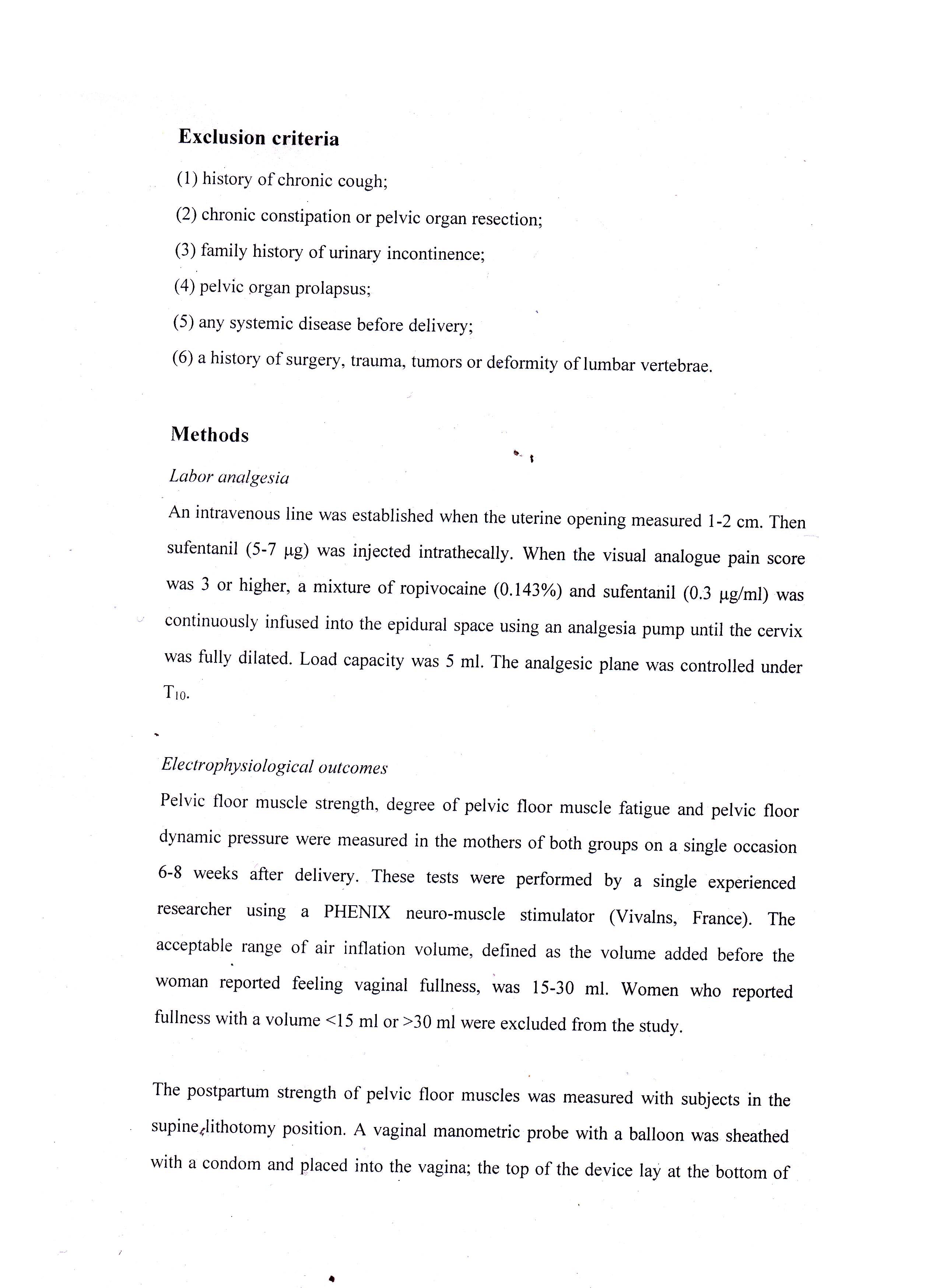


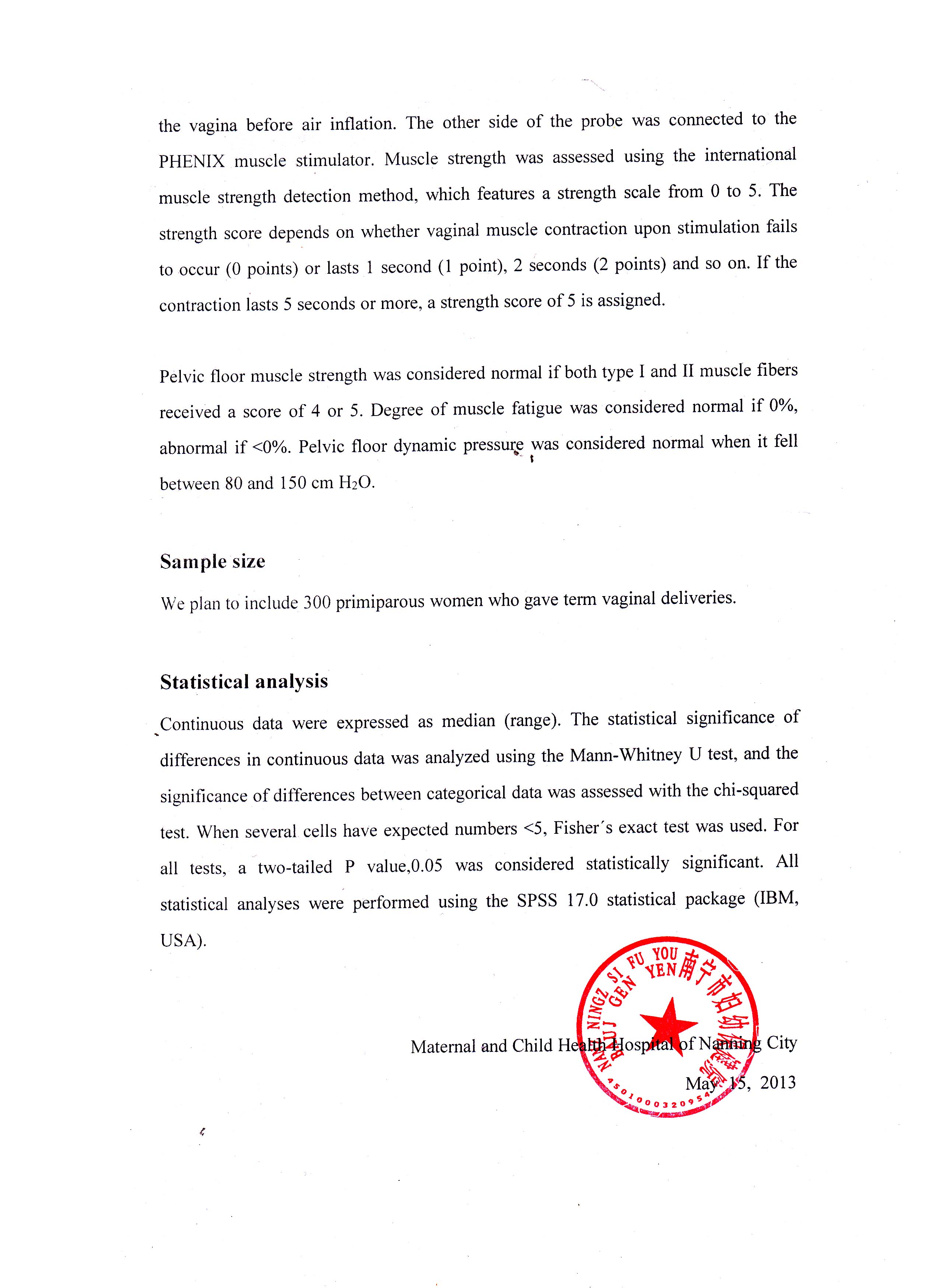

Supplement: S1 Protocol — (DOC) [file pone.0137267.s002.doc]

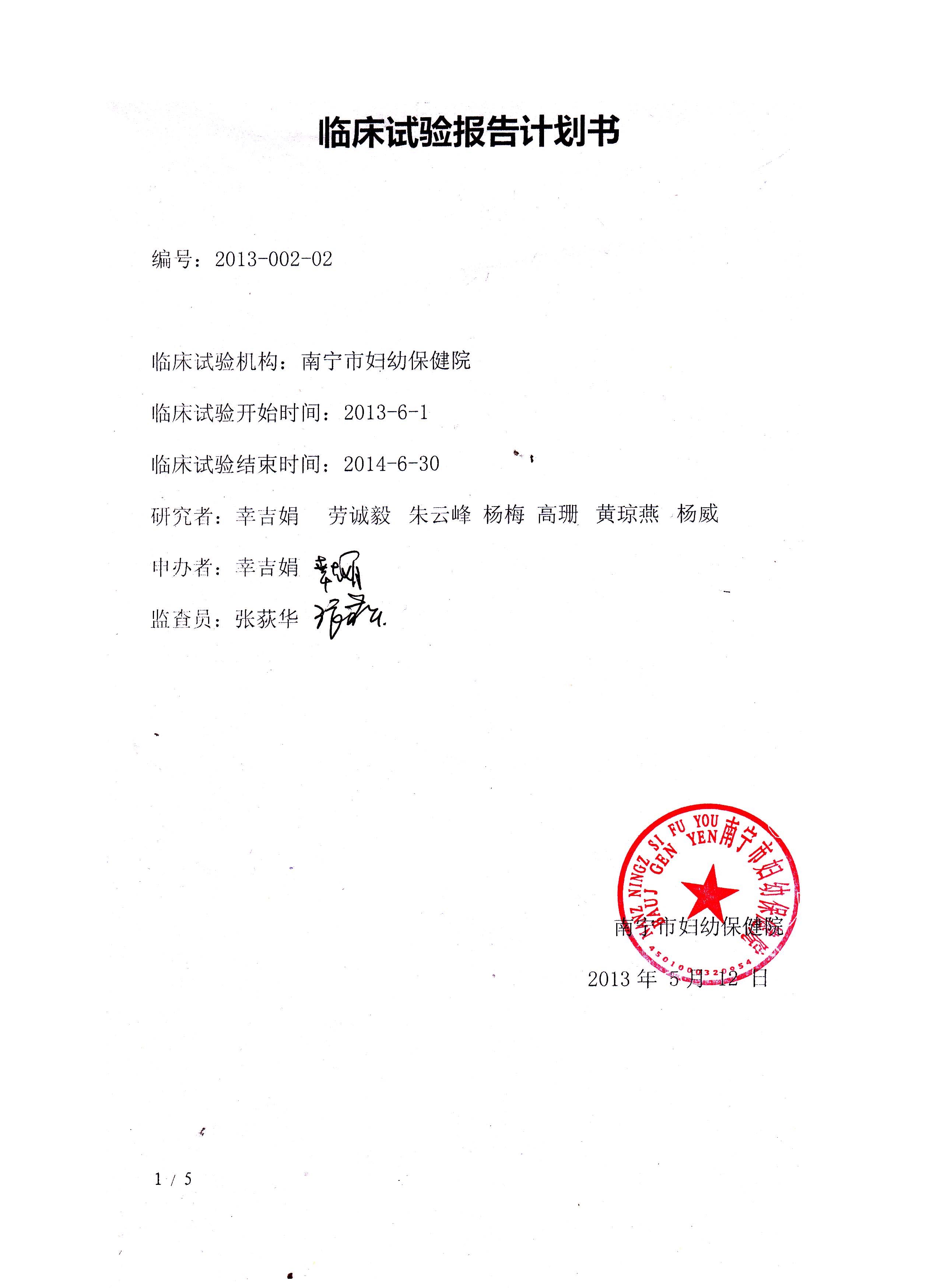


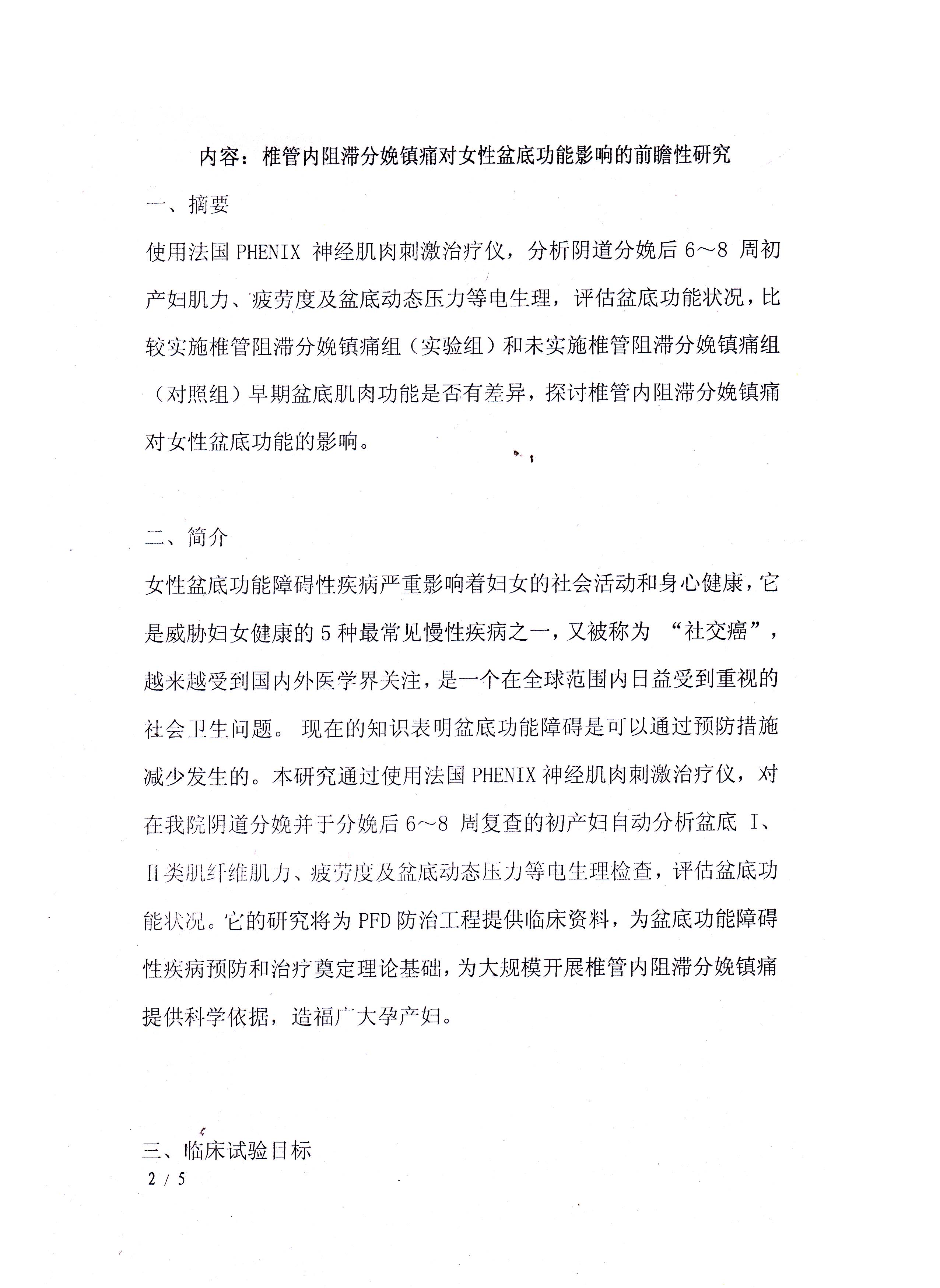


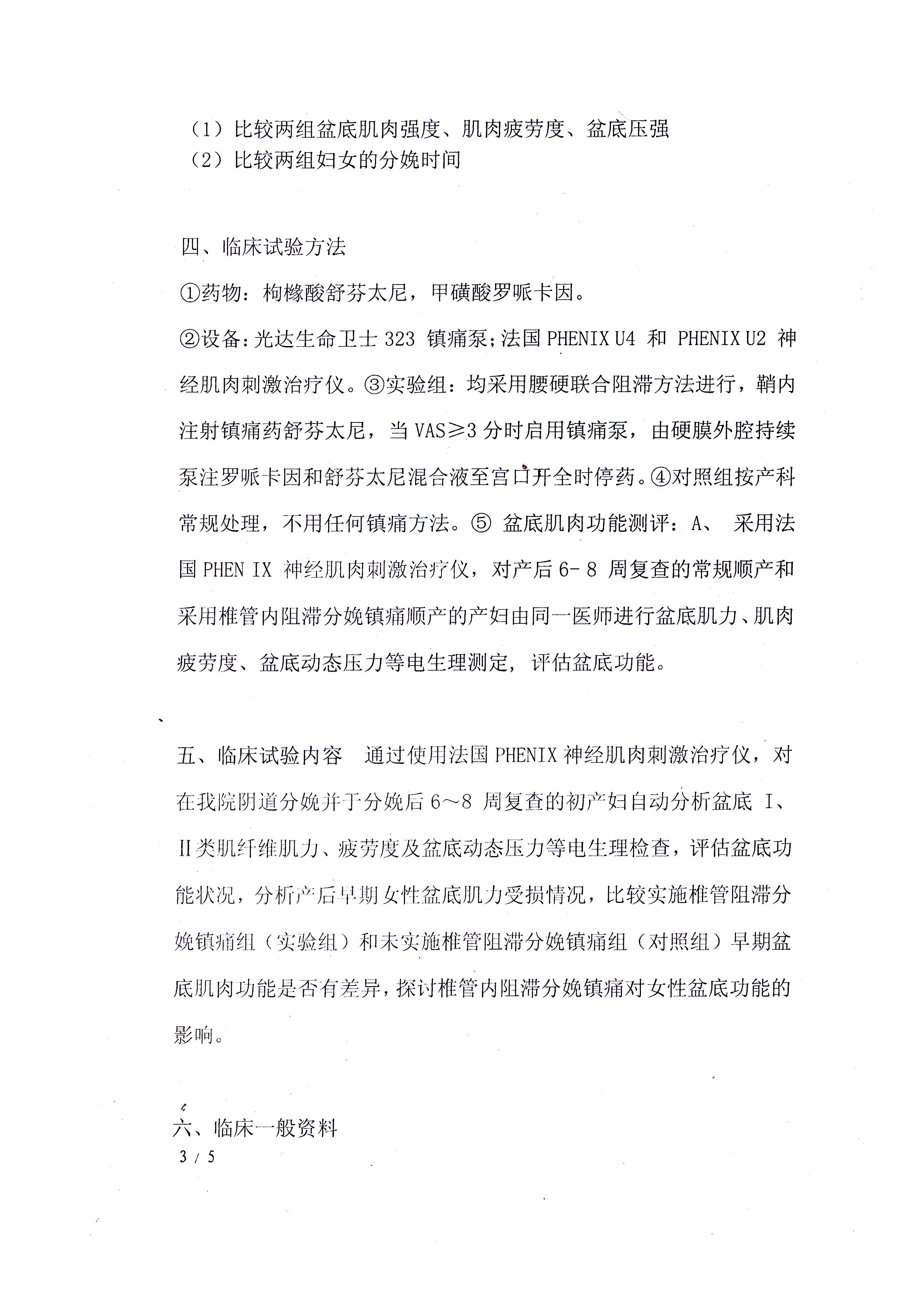


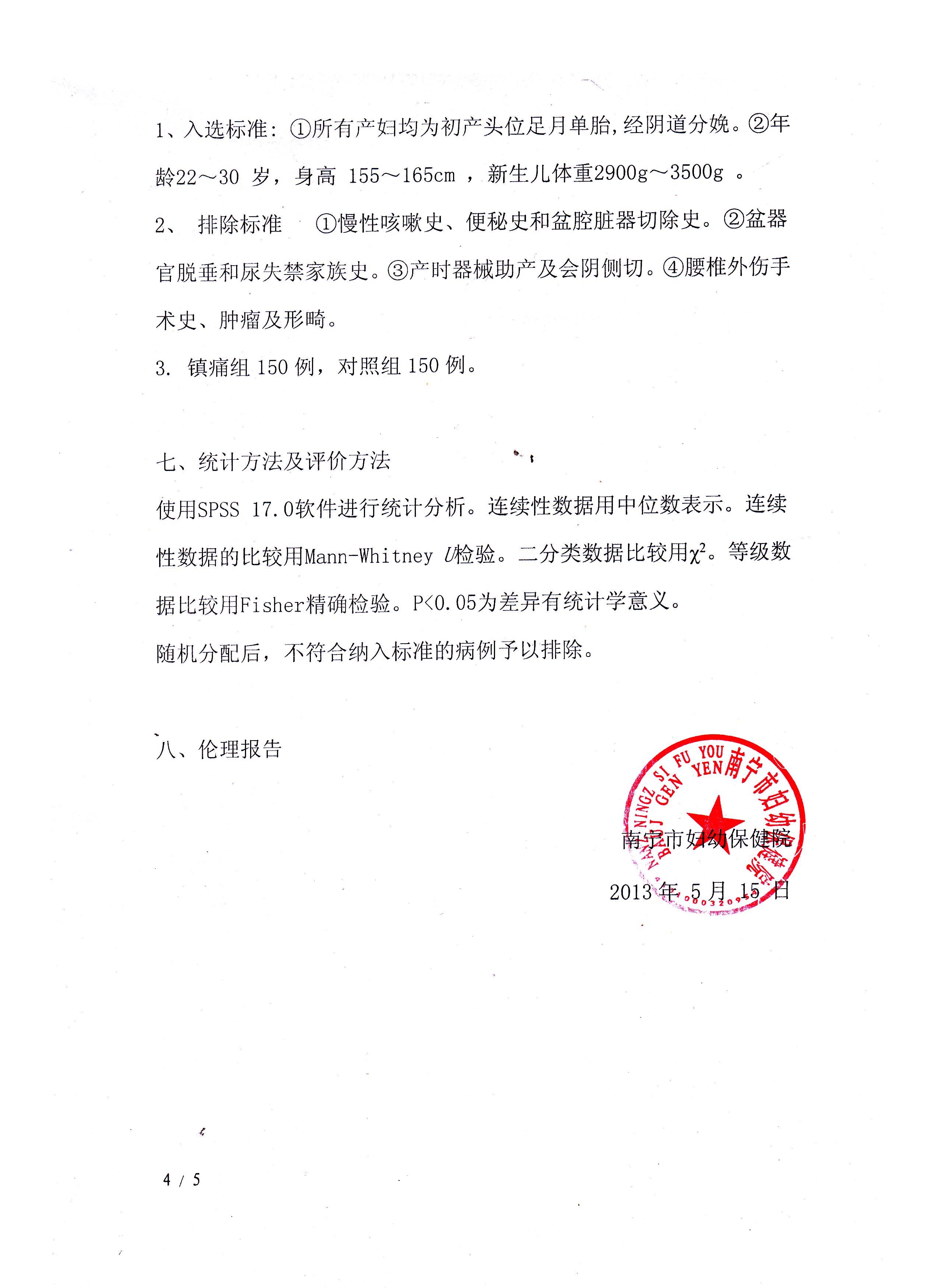


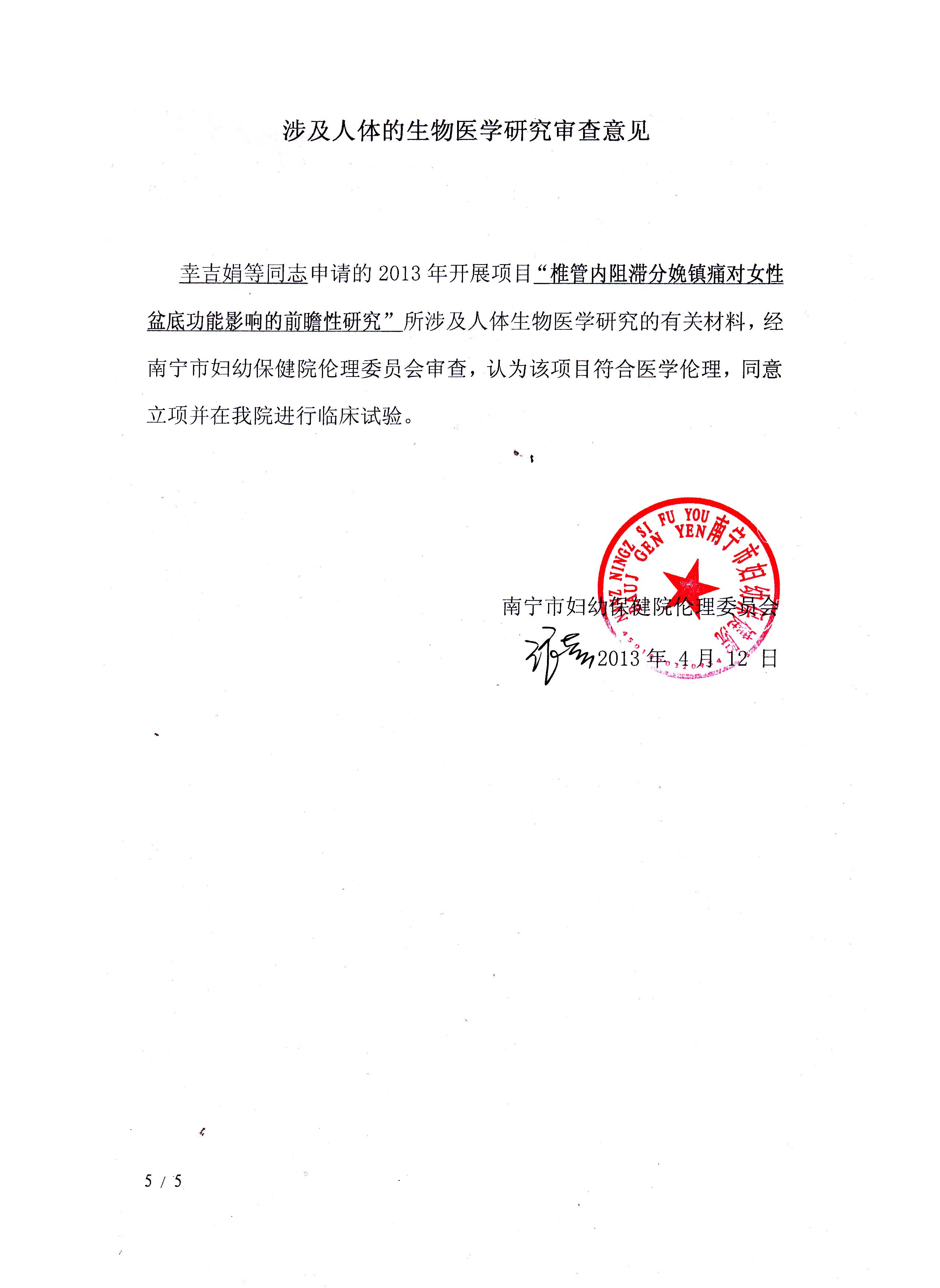

Supplement: S2 Protocol — (DOC) [file pone.0137267.s003.doc]
